# Network pharmacology and molecular docking study on the mechanism of colorectal cancer treatment using Xiao-Chai-Hu-Tang

**DOI:** 10.1371/journal.pone.0252508

**Published:** 2021-06-14

**Authors:** Jingyun Jin, Bin Chen, Xiangyang Zhan, Zhiyi Zhou, Hui Liu, Yun Dong

**Affiliations:** 1 Department of Oncology, Shuguang Hospital, Shanghai University of Traditional Chinese Medicine, Shanghai, China; 2 Shanghai University of Traditional Chinese Medicine, Shanghai, China; 3 Department of Oncology, Putuo Hospital, Shanghai University of Traditional Chinese Medicine, Shanghai, China; 4 Department of Oncology, Longhua Hospital, Shanghai University of Traditional Chinese Medicine, Shanghai, China; 5 Department of Integrated Traditional Chinese Medicine and Western Medicine, Shanghai Chest Hospital, Shanghai Jiaotong University, Shanghai, China; University of Pittsburgh, UNITED STATES

## Abstract

**Background and objective:**

We aimed to predict the targets and signal pathways of Xiao-Chai-Hu-Tang (XCHT) in the treatment of colorectal cancer (CRC) based on network pharmacology, just as well as to further analyze its anti-CRC material basis and mechanism of action.

**Methods:**

We adopted Traditional Chinese Medicine Systems Pharmacology Database (TCMSP) and Traditional Chinese Medicine Integrated Database (TCMID) databases to screen the active ingredients and potential targets of XCHT. CRC-related targets were retrieved by analyzing published microarray data (accession number GSE110224) from the Gene Expression Omnibus (GEO) database. The common targets were used to construct the “herb-active ingredient-target” network using the Cytoscape 3.8.0 software. Next, we constructed and analyzed protein-to-protein interaction (PPI) using BisoGenet and CytoNCA plug-in in Cytoscape. We then performed Gene Ontology (GO) functional and the Kyoto Encyclopaedia of Genes and Genomes (KEGG) pathway enrichment analyses of target genes using the R package of clusterProfiler. Furthermore, we used the AutoDock Tools software to conduct molecular docking studies on the active ingredients and key targets to verify the network pharmacological analysis results.

**Results:**

We identified a total of 71 active XCHT ingredients and 20 potential anti-CRC targets. The network analysis revealed quercetin, stigmasterol, kaempferol, baicalein, and acacetin as potential key compounds, and PTGS2, NR3C2, CA2, and MMP1 as potential key targets. The active ingredients of XCHT interacted with most CRC disease targets. We showed that XCHT’s therapeutic effect was attributed to its synergistic action (multi-compound, multi-target, and multi-pathway). Our GO enrichment analysis showed 46 GO entries, including 20 biological processes, 6 cellular components, and 20 molecular functions. We identified 11 KEGG signaling pathways, including the IL-17, TNF, Toll-like receptor, and NF-kappa B signaling pathways. Our results showed that XCHT could play a role in CRC treatment by regulating different signaling pathways. The molecular docking experiment confirmed the correlation between five core compounds (quercetin, stigmasterol, kaempferol, baicalein, and acacetin) just as well as PTGS2, NR3C2, CA2, and MMP1.

**Conclusion:**

In this study, we described the potential active ingredients, possible targets, and key biological pathways responsible for the efficacy of XCHT in CRC treatment, providing a theoretical basis for further research.

## Introduction

Colorectal cancer (CRC) is the third most common global malignancy in the world and the fourth leading cause of cancer-related death [1]. In recent years, the incidence and mortality rates of colorectal cancer have increased significantly in developing countries [2]. At present, CRC treatment mainly comprises surgery, radiotherapy, and chemotherapy with Traditional Chinese Medicine (TCM). TCM has been widely accepted worldwide as an essential complementary medicine that provides beneficial therapeutic effects for cancer patients [3]. In United States of America(USA), Chinese herbal medicine has been used as an auxiliary medicine for cancer treatment [4]. Previous studies have shown that traditional herbal medicine as an adjuvant therapy, combined with chemotherapy or radiation therapy, could improve the therapeutic effects and quality of life, reduce adverse effects, and prolong survival [5–9].

Xiao-Chai-Hu-Tang (XCHT), derived from the Treatise on Febrile and Miscellaneous Diseases, is an extract of seven herbs: *Radix bupleuri* (Chai-Hu), *Pinellia ternata* (Ban-Xia), *Scutellaria baicalensis* (Huang-Qin or Chinese skullcap root), *Zizyphus jujube* (DA-Zao or jujube fruit), *Panax ginseng* (Ren-Shen or ginseng root), *Zingiber officinale* (Sheng-Jiang or ginger rhizome), and *Prepared Liquorice Root* (Zhi-Gan-Cao). Previous studies showed that XCHT could treat tumors by enhancing immune regulation, anti-angiogenesis, and tumor cell apoptosis [10, 11]. XCHT and its plus or minus formula exhibit an excellent experimental and clinical achievements in malignant tumors such as gastric [12], lung [13], breast [14], and liver cancer [15], and it also has a good prospect in CRC treatment. Certain studies showed [16] that XCHT might inhibit CRC progression in a chronic stress mouse CRC model by inhibiting the NLRP3 inflammasome activity and pro-inflammatory factor secretion, just as well as by down-regulating NF-κB. However, the specific underlying mechanisms of these effects are still unclear.

**Fig 1:**
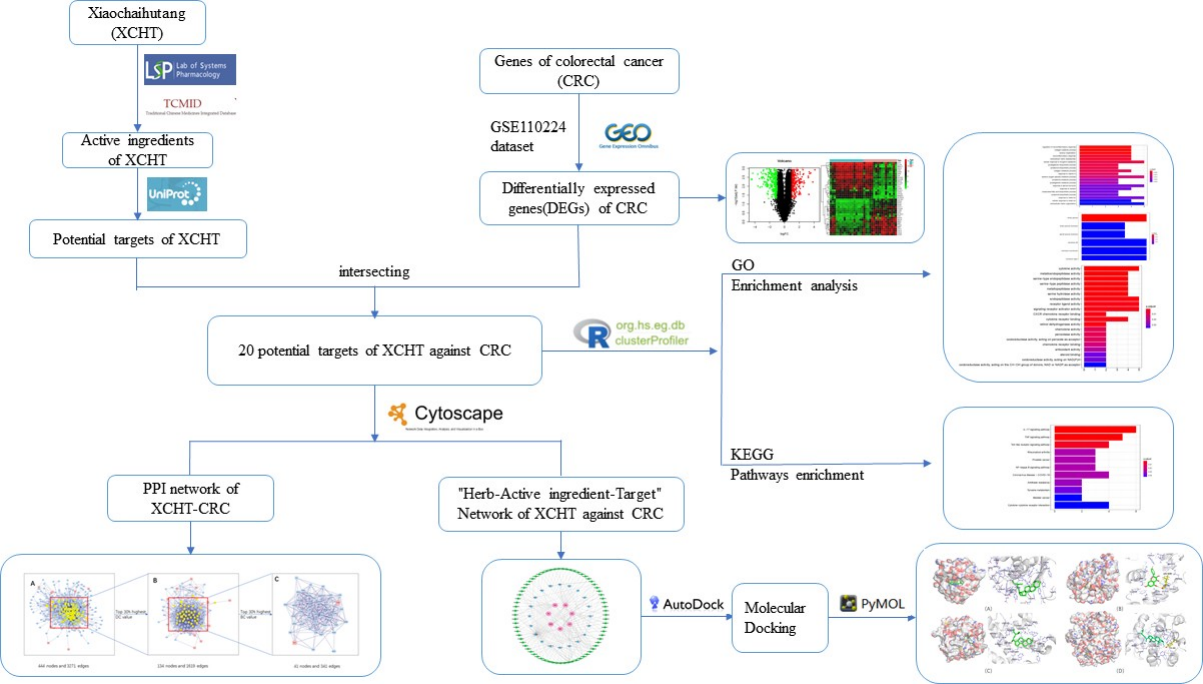
Research process.

Network pharmacology is a network viewpoint-based new approach to identify the effector mechanism of herb-ingredient-target multimolecular synergy at the systemic level [17]. Therefore, this study aimed at predicting the XCHT target and signaling pathways against CRC from a network pharmacology aspect and further analyzing the anti-CRC potential and effector mechanism of XCHT. Moreover, we used molecular docking technology for verification, to provide a theoretical foundation for further studies and reasonable clinical applications of XCHT against CRC (Fig 1).

## Materials and methods

### XCHT chemical compounds and targets

We obtained the XCHT active ingredients and corresponding targets from the TCMSP(http://lsp.nwu.edu.cn/, Ver.2.3) and the TCMID (http://www.megabionet.org/tcmid/, Ver.1.0) using the keywords of "ChaiHu", "HuangQin", "BanXia", "DaZao", "ShengJiang", "RenShen" and "ZhiGanCao". In this study, oral bioavailability (OB) and drug-likeness (DL) were adopted to identify the bioactive ingredients of XCHT. Only compounds with related targets that met the absorption, distribution, metabolism, excretion (ADME) criteria (ie, OB threshold > 30% and DL threshold > 0.18) were included in the study for subsequent research. Next, restricting the species to humans, the Uniprot KB function of Uniprot (http://www.uniprot.org/)database was used to retrieve and match targets of compounds. Compounds without corresponding targets and redundant duplicates were removed. Eventually, the targets of active components were obtained and converted into gene symbols.

### Identifying differentially expressed CRC genes

The microarray expression profile dataset GSE110224 was downloaded from the National Center for Biotechnology Information (NCBI) GEO (http://www.ncbi.nlm.nih.gov/geo/) database, which is based on the GPL570 [HG-U133_Plus_2] Affymetrix Human GenomeU133 Plus 2.0 Array platform. The dataset contained 34 samples, including 17 primary colorectal cancer tissue samples and 17 matched normal tissue samples. The script was run using the Strawberry Perl-5.30.2.1 (Perl) software, and the gene probe names were annotated as gene symbols and grouped. Then, R packages (“limma” and “pheatmap”) were used to analyze GSE110224. Differentially expressed genes (DEGs) between the normal and colorectal cancer tissues were detected on the basis of the criteria adjusted P-value of *p* < 0.05 and |logic| > 1 and were visualized using a volcano plot.

### Herb-active ingredient-target interaction network construction

The XCHT action targets and the CRC DEGs were taken as an intersection, and the intersection targets of these two parts were defined as the key targets of XCHT in the CRC treatment. The herb-active ingredient-target interaction network was visualized using the Cytoscape 3.8.0 software (www.cytoscape.org/). Taking disease, key targets, herbs, active ingredients as nodes, the corresponding relationship above all was constructed with Microsoft Excel, then imported into Cytoscape 3.8.0 to construct the interaction network of XCHT in the treatment of CRC. Then, the Analyze Network plug-in in Cytoscape was used to calculate and sort the topological parameters (degree) of the network.

### Construction and analysis of the protein-protein interaction (PPI) network

The PPI network was constructed using the BisoGenet plug-in in Cytoscape. CytoNCA, a Cytoscape plugin for network centrality analysis, was used to identify crucial genes in the network. First, Genes with the highest degree centrality (DC) values in the top 30% were selected for subnetwork construction using CytoNCA. Then, genes with the top 30% highest value of betweenness centrality (BC) in the subnetwork were identified as key genes and formed the core network.

### GO and KEGG pathway enrichment

Go biological function analysis is mainly used to describe the functions of gene targets, including molecular function (MF), cellular components (CC), and biological processes (BP). KEGG enrichment analysis allows the obtention of the signaling pathways enriched by the common targets of XCHT and CRC. GO and KEGG Pathways Enrichments were constructed using the R packages (“clusterProfiler,” “org. Hs. Aug. deb,” “enrichplot,” and “ggplot2”) based on the p-value cutoff = 0.05 and q-value cutoff = 0.05 criteria.

### Molecular docking

Molecular docking was performed using AutodockTools-1.5.6. The atomic coordinates of the crucial target were retrieved from the Protein Data Bank (PDB) (www.rcsb.org) and prepared in AutoDockTools-1.5.6 by removing water molecules, adding charge, and parameterizing. The 3D structures of active ingredients were downloaded from the TCMSP Database and prepared in AutoDockTools by computing atomic partial charges and parameterizing. The docking site was set in a cubic box in the center of the initial ligand, and a grid map of each atom type in the box was computed. The AutoDockTools-1.5.6 software was used to simulate the molecular docking of potential targets and components. The best scoring conformer of each compound was analyzed and visualized in AutoDockTools-1.5.6 and PYMOL.

## Results

### XCHT active ingredients and potential targets

In this study, the TCMSP database was used to search for all XCHT compounds and 124 active compounds were selected based on the OB > 30% and DL > 0.18 criteria. Corresponding relationships between the active ingredients and the targets, deleting duplicate targets, and converting targets into Gene Symbol using Uniprot, 82 active compounds and 238 targets were obtained after removing the chemical compounds that lacked potential target information. We identified 12, 32, 2, 17, 11, 4, and 18 active compounds for *Radix bupleuri*, *Scutellaria baicalensis*, *Prepared Liquorice Root*, *Panax ginseng*, *Pinellia ternata*, *Zingiber officinale*, and *Zizyphus jujube*, respectively (S1 Table).

### CRC DEG screening

**Fig 2:**
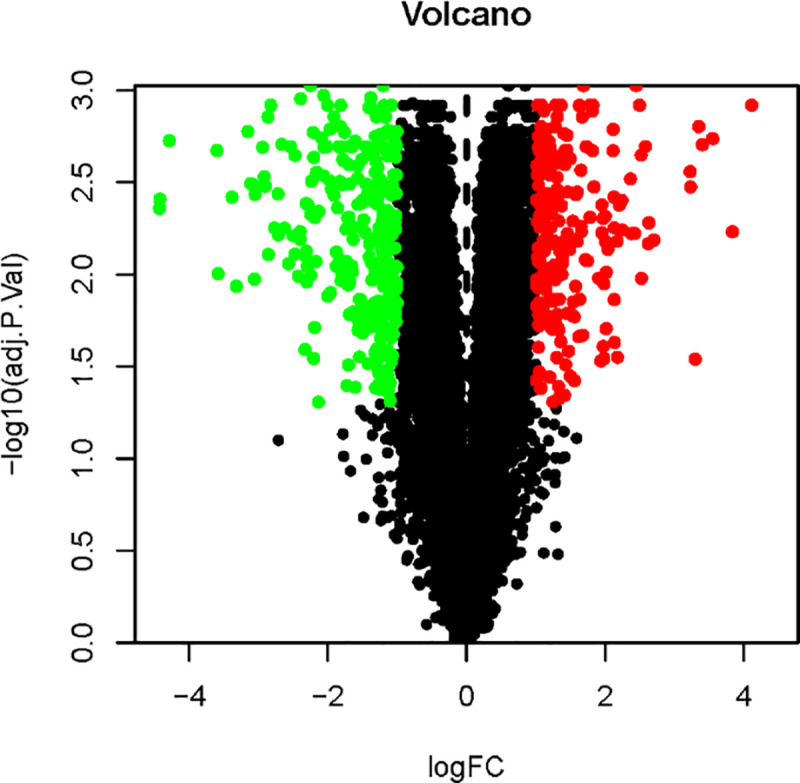
Volcano map of differentially expressed genes. The red and the green dots represent DEGs that were significantly upregulated (genes) and downregulated (genes), respectively (adjusted p-value < 0.05, and |logic| > 1).

**Fig 3:**
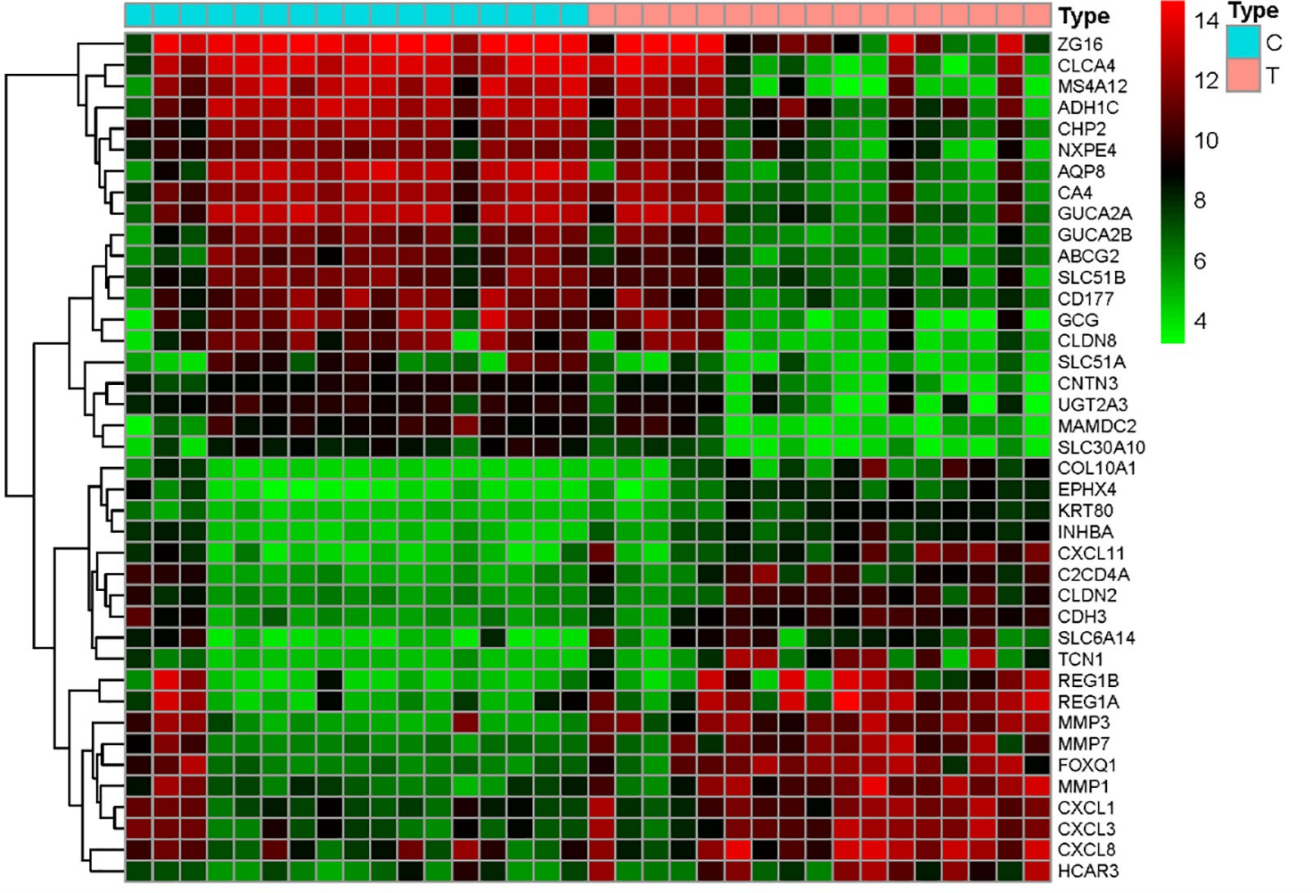
Heatmap of differentially expressed genes. Red indicates that the gene is highly expressed in the sample, and green indicates that the gene has a lower expression in the sample.

The analysis of the GEO database-derived GSE110224 dataset yielded 519 DEGs based on the cutoff criteria, including 296 downregulated and 223 upregulated genes. The volcano map and DEG distribution heatmap are shown in Figs 2 and 3, respectively. The top 10 significantly upregulated and downregulated DEGs are listed in Table 1.

**Table 1:** The identified top 10 upregulated and downregulated DEGs between CRC tissue samples and normal tissue samples.

	Upregulated DEGs			Downregulated DEGs	
Gene name	Logic	Adjusted P value	Gene name	Logic	Adjusted P value
MMP3	4.1151	1.21E-03	CLCA4	-4.4301	4.39E-03
REG1A	3.8374	5.87E-03	MS4A12	-4.4268	3.91E-03
FOXQ1	3.5542	1.83E-03	AQP8	-4.2887	1.88E-03
CXCL11	3.4058	1.98E-03	CA4	-3.6018	2.13E-03
MMP7	3.3532	1.58E-03	GCG	-3.5859	9.92E-03
REG1B	3.3007	2.89E-02	GUCA2A	-3.3854	3.80E-03
TCN1	3.2331	3.36E-03	CLDN8	-3.3201	1.16E-02
MMP1	3.2274	2.77E-03	CHP2	-3.1559	1.68E-03
SLC6A14	2.7038	6.51E-03	NXPE4	-3.1142	3.22E-03
CXCL3	2.6319	5.25E-03	MAMDC2	-3.0747	3.26E-03

### XCHT " Herb-active ingredient-target " network construction and analysis

**Fig 4:**
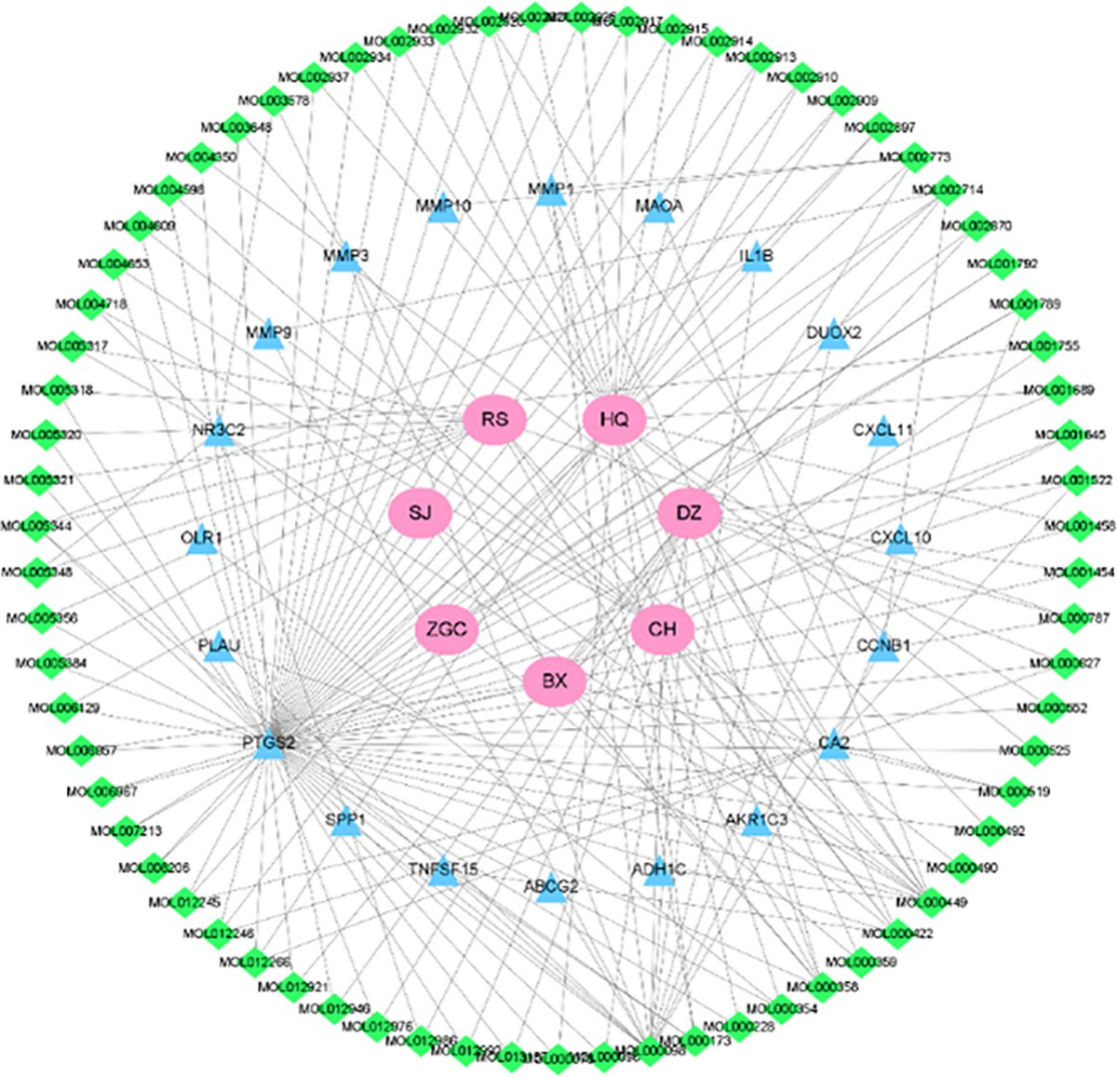
"Herb-active ingredient-target" network of XCHT against CRC.

After intersecting the 238 XCHT-related targets with 519 CRC DEGs, 20 genes were acquired as crucial anti-CRC XCHT genes (S2 Table). The anti-CRC XCHT "Herb-Active ingredient-Target" Network was constructed using the Cytoscape software (Fig 4), containing 98 nodes (71 compounds, 20 target genes, and 7 herb nodes) and 191 edges of the network, of which pink circles stand for the XCHT herbs, green quadrangles for active ingredients, and the blue diamonds for potential targets. The links between the nodes represent the functional relationships of these nodes. The higher the number of the connected nodes, the more critical the role of the targets or compounds becomes in this network. Next, we calculated the network degree using Analyze-Network. We discovered that quercetin, stigmasterol, kaempferol, baicalein, and acacetin were the top 5 in terms of the degree of all the active ingredients. Moreover, PTGS2 was the gene associated with the highest number of active ingredients, followed by NR3C2, CA2, MMP1, TNFSF15, and CCNB1 (S3 Table).

Fig 4 shows the interactions between the active XCHT ingredients and most CRC targets, fully illustrating that XCHT exerts a therapeutic effect through the synergistic actions of multiple compounds, targets, and pathways. Among them, quercetin and stigmasterol exhibited more interactions, suggesting that these active ingredients play a crucial role in the anti-CRC effector mechanism of XCHT.

### CRC target PPI network analysis

**Fig 5:**
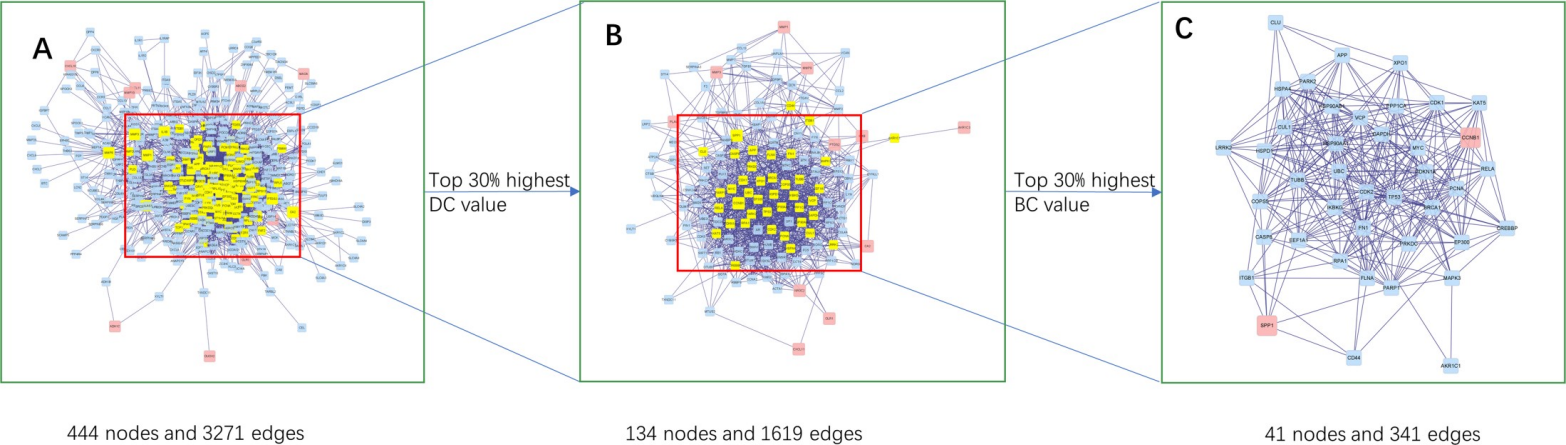
Topological analysis of the protein-protein interaction network. (A) The PPI network of XCHT-CRC target genes. (B) The PPI network of significant proteins extracted from A. (C) The PPI network of crucial XCHT targets for CRC treatment extracted from B.

The XCHT-CRC target gene PPI network comprised 444 nodes and 3,271 edges (Fig 5A), and the subnetwork was acquired through selecting the top 30%-degree centrality (DC), consisting of 134 nodes and 1,619 edges (Fig 5B). After screening the top 30%-betweenness centrality (BC) of the subnetwork, the core network was constructed, including 41 nodes and 341 edges (Fig 5C). Based on the core network, two target genes, CCNB1 and SPP1, were defined as the critical genes in the PPI core network, further verifying the crucial role of the two proteins.

### GO and pathway enrichment analyses

**Fig 6:**
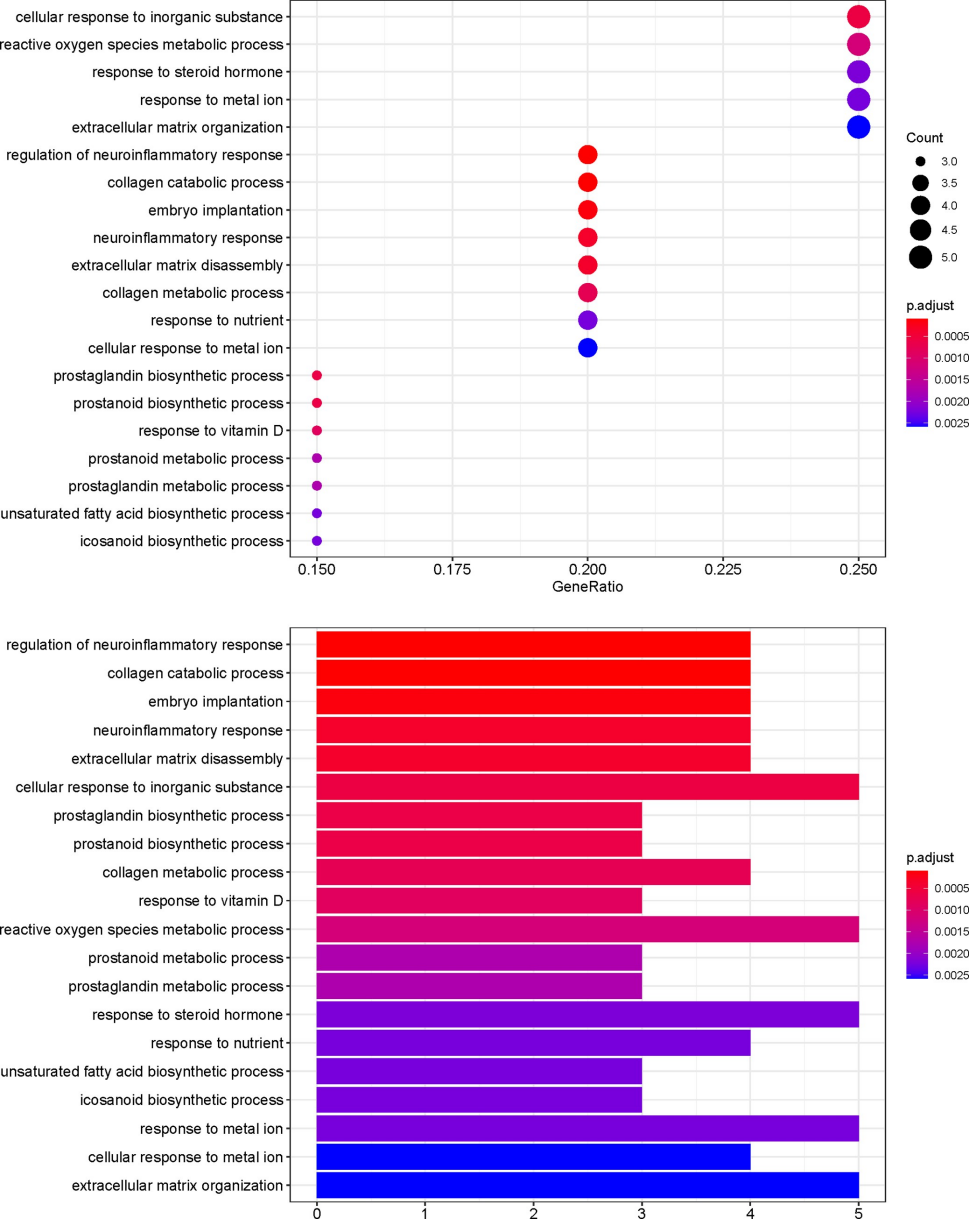
BP analysis of GO enrichment.

**Fig 7:**
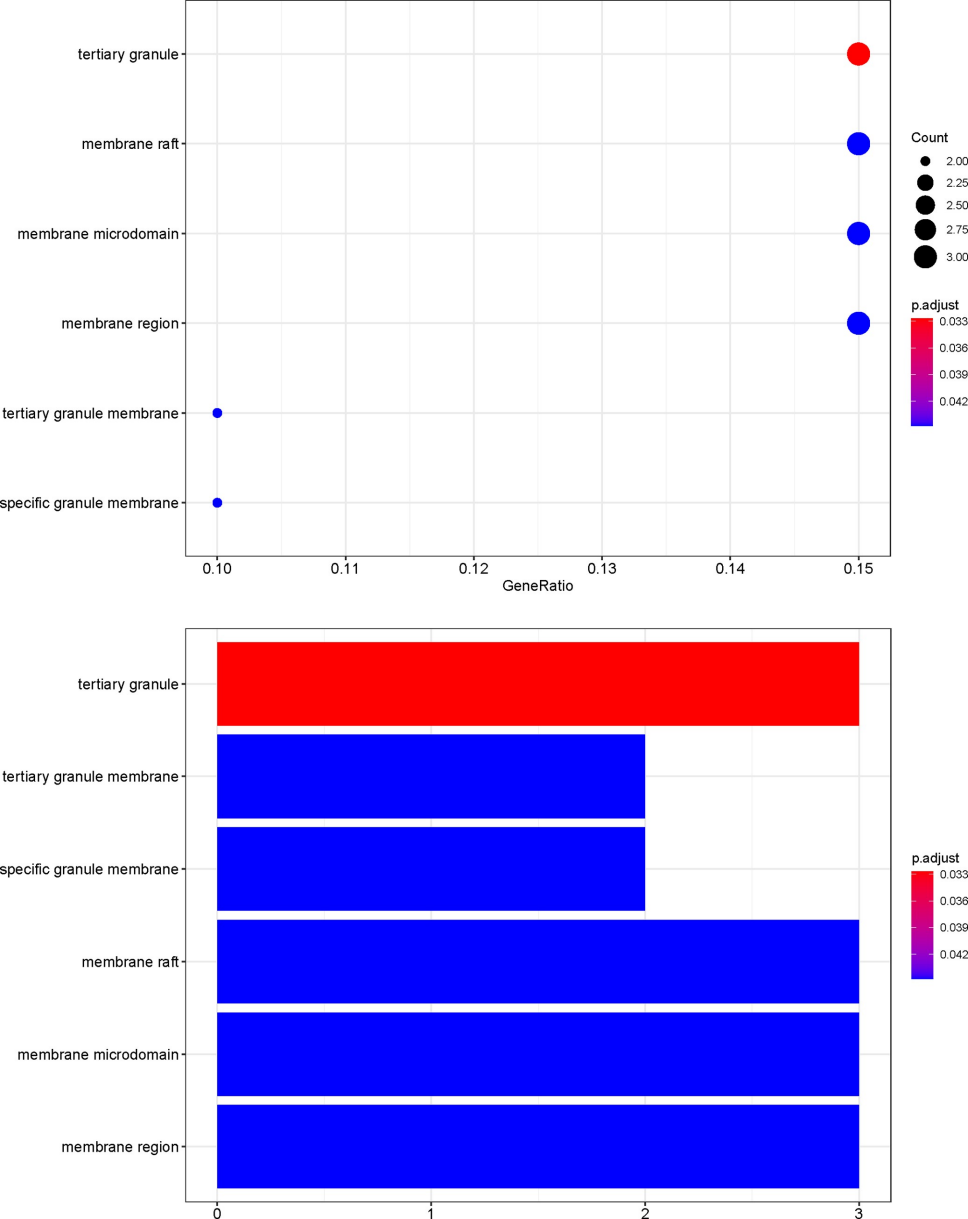
CC analysis of GO enrichment.

**Fig 8:**
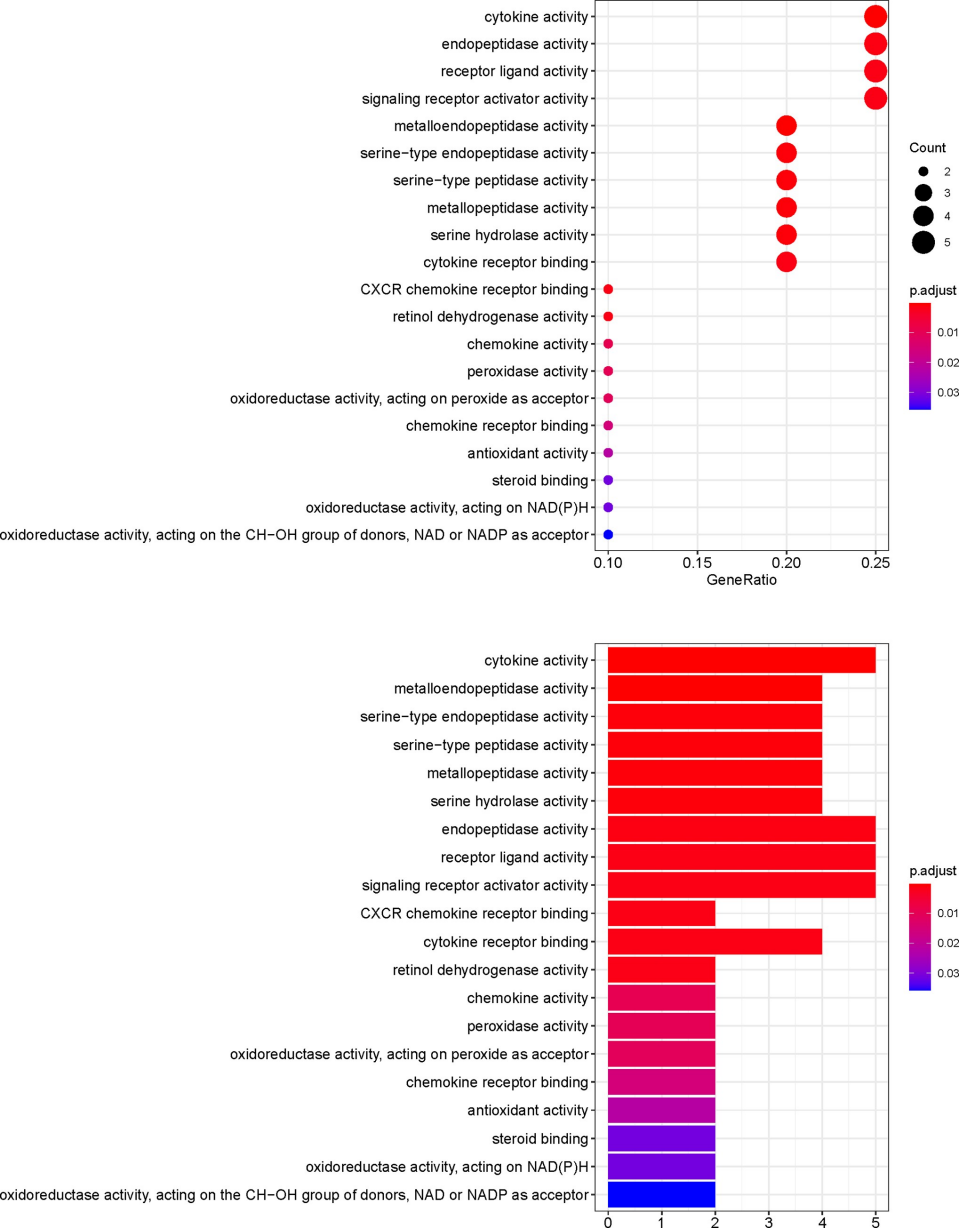
MF analysis of GO enrichment.

**Fig 9:**
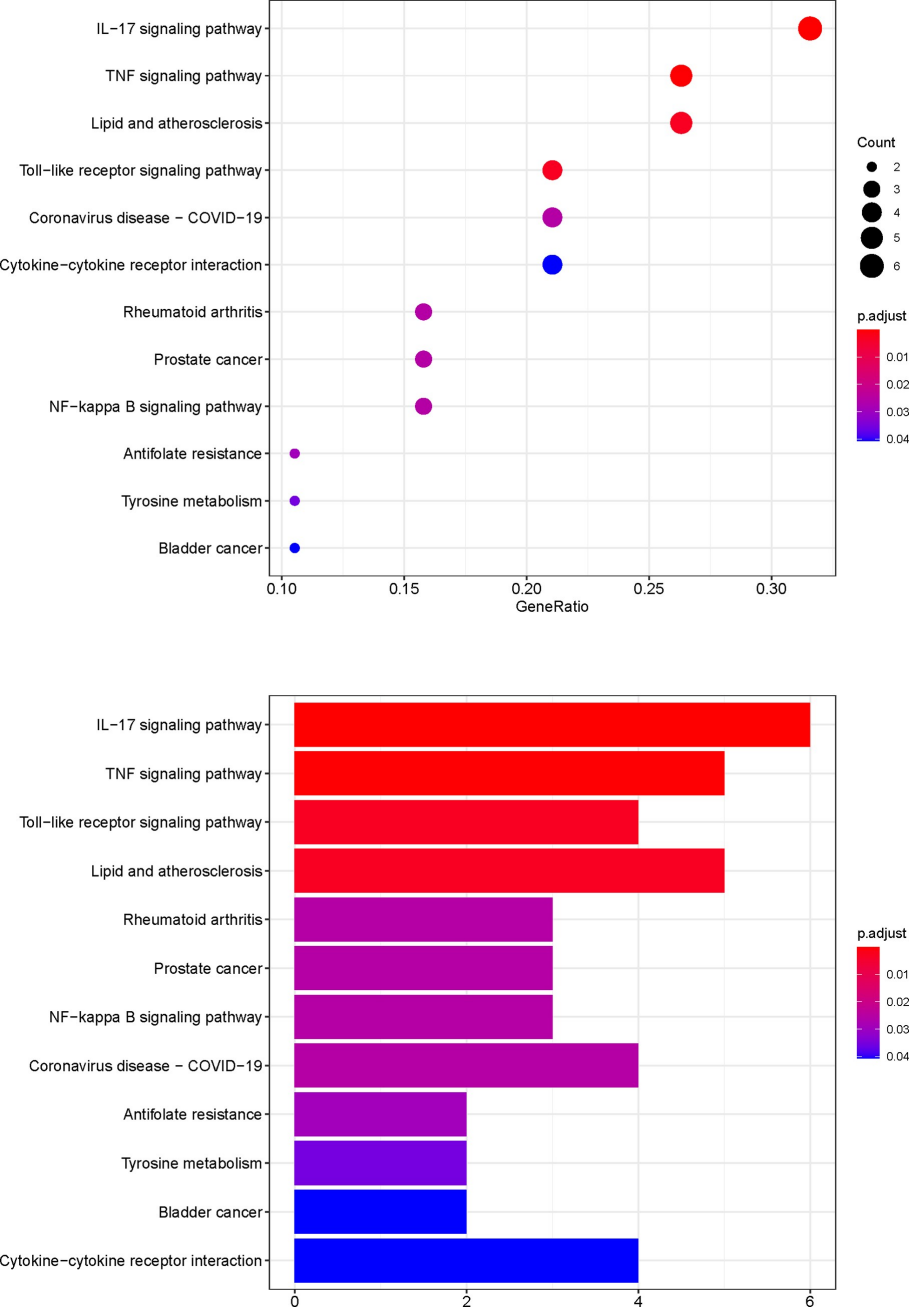
KEGG pathway enrichment analysis. The redder the color of the term, the smaller its P-value.

To further investigate the effector mechanism of XCHT in the treatment of CRC, the crucial target genes were analyzed by GO biological process enrichment and KEGG pathway enrichment analysis. The GO enrichment analysis comprised three parts: biological process (BP), cellular components (CC), and molecular function (MF). 20 potential XCHT targets for CRC were analyzed using R packages. The top 20 terms of BP and MF, just as well as 6 terms of CC are shown in Figs 6–8 and S4 Table. The top 11 pathways of the KEGG enrichment analysis are shown in Fig 9 and S5 Table.

Among Figs 6–9, the bubble graphs are on the left and the bookplates are on the right. In the bar plots below, each bar represents a GO and KEGG term on the vertical axis. The number of genes enriched in each term is represented on the horizontal axis. The color of each bar represents the adjusted p-value of each GO and KEGG term. The redder the color of the term, the smaller its adjusted p-value is. Similarly, in the bubble graphs below, each bubble represents a GO and KEGG path on the vertical axis. The proportion of the genes is represented on the horizontal axis. The size of each bubble indicates the number of genes enriched in each GO and KEGG pathway. The larger the bubble, the greater is the number of genes involved in the pathway. The color of each bubble represents the adjusted P-value for each GO and KEGG path. The redder the bubble, the smaller the adjusted P-value is.

**Fig 10:**
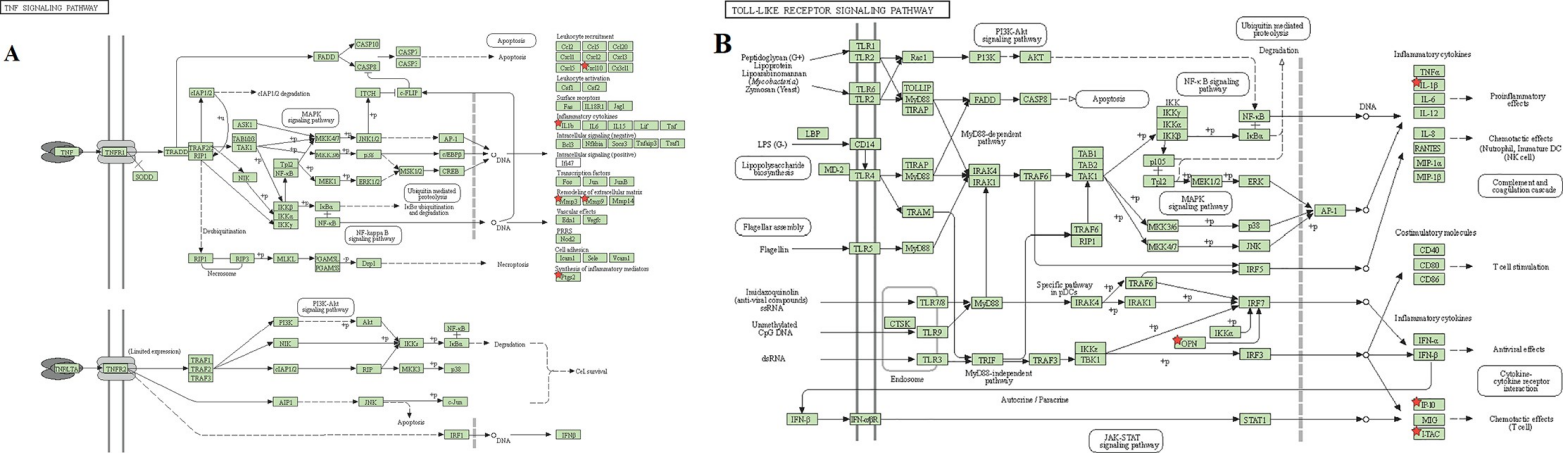
KEGG enrichment diagram. (A) TNF signaling pathway. (B) Toll-like receptor signaling pathway. Red asterisks represent core targets.

The top 11 pathways of the KEGG enrichment analysis are shown in Fig 9, such as the IL-17, TNF (Fig 10A), Toll-like receptor (Fig 10B), or NF-kappa B signaling pathways. The targets of the active XCHT ingredients were distributed in different signaling pathways, coordinated with each other, and exerted their anti-CRC therapeutic effect by regulating the different signaling pathways.

### Molecular docking

Five active ingredients (quercetin, stigmasterol, kaempferol, baicalein, and acacetin) and four potential target genes (PTGS2, NR3C2, CA2, and MMP1) were selected through network analysis, with high node scores and confidence. Molecular docking studies were conducted to identify the interactions between the active XCHT ingredients and CRC-related potential target genes at the molecular level. The AutoDockTools-1.5.6 software was used for molecular docking. The docking score is shown in Table 2. The docking site was set in a cubic box in the center of the initial ligand. The lower the docking score (the greater the negative value), the higher the binding force is between the compound and the protein. The molecular docking results showed that the docking scores of quercetin, stigmasterol, kaempferol, baicalein, and acacetin to the PTGS2, NR3C2, CA2, and MMP1 proteins were ≤ -5.0 KJ/Mol, suggesting that these compounds could exert a strong binding effect with the key proteins.

**Table 2:** The docking affinity and interactions of compounds binding to key targets.

ID	Compound	Affinity (kcal/Mol)			
		PTGS2	NR3C2	CA2	MMP1
MOL000098	quercetin	-6.71	-7.49	-7.11	-8.27
MOL000449	Stigmasterol	-9.61	-9.97	-7.16	-10.18
MOL000422	Kaempferol	-7.42	-9.02	-6.11	-8.59
MOL002714	Baicalein	-6.8	-7.64	-5.4	-8.41
MOL001689	Acacetin	-8.29	-8.95	-5.07	-8.58
CAS:51-21-8	5-FU	-4.55	-4.09	-5.18	-4.59

**Fig 11:**
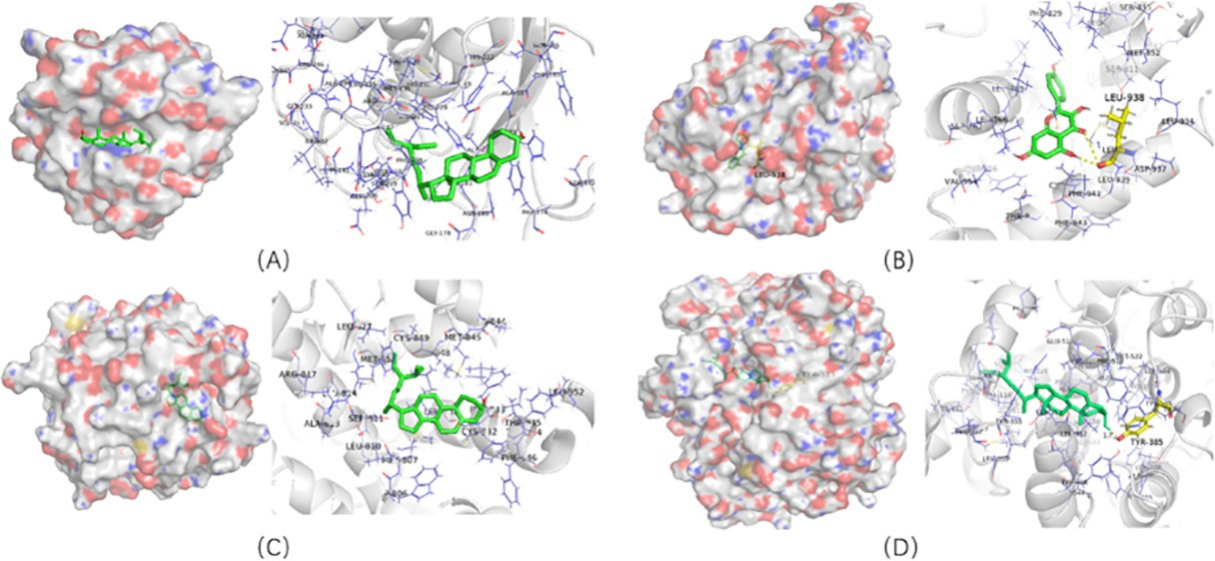
Part of molecular docking results. (A). Stigmasterol—MMP1; (B). kaempferol—NR3C2; (C). Stigmasterol—NR3C2; (D). Stigmasterol—PTGS2. The compound structure was presented as a green stick, the protein structure was presented as a gray ribbon and the interacted residues were shown as a red and blue lines respectively.

In addition, 5-FU, a positive control drug, was the main drug for colorectal cancer chemotherapy. Studies have shown that the anti-tumor effect of 5-FU has a certain correlation with PTGS2, NR3C2, CA2 and MMP1 [18–22]. We observed that the 5-FU target docked with the key proteins, and the obtained affinity data were used as the baseline of the positive control. According to the molecular docking results (S1 Fig), all 5 active components of XCHT exhibited good binding properties to the key targets, and their binding energies were all less than that of two 5-FU, suggesting that these compounds could bind stably to the active pockets of the PTGS2, NR3C2, CA2, and MMP1 proteins. Selecting 4 target groups with a strong affinity to construct 3D diagrams of molecular docking (Fig 11).

## Discussion

XCHT is derived from the Treatise on Febrile and Miscellaneous Diseases. Previous studies have shown its treatment potential targeting various malignant tumors, including CRC, but the detailed underlying effector mechanism remains unclear.

In this study, we analyzed the "herb-active ingredient-target" network and found that quercetin, stigmasterol, kaempferol, baicalein, and acacetin had a high correlation in the network, exhibiting significant anti-colon cancer effects [23–26]. For example, quercetin could inhibit CRC by inhibiting the cell cycle, angiogenesis, and metastasis or increasing apoptosis [23]. Previous studies have shown that baicalein could inhibit MMP-1 expression [27], and our study verified that bicycling and MMP-1 exhibit high binding energy through molecular docking. However, the downregulation of MMP‑1 expression inhibited the progression of colorectal cancer [28], it could thus be speculated that baicalein could slow down the progression of colorectal cancer by inhibiting MMP-1 expression. Kaempferol exhibits strong cytotoxic, anti-oxidant, anti-proliferative, and anti-apoptotic effects against HCT-116, HCT-15, SW480, and other CRC cells [29], and could also be used alone or combined with 5-FU to overcome CRC drug resistance [30]. Acacetin induces mitochondrial ROS-mediated cell death in a caspase-independent manner in SW480 and HCT-116 colon carcinoma cells by inducing an apoptosis-inducing factor (AIF) to enhance the therapeutic effect of anti-CRC [31], which may be the material basis of XCHT in the treatment of CRC.

PTGS2, also known as COX-2, is typically induced by inflammatory stimuli and expressed at high levels in Colorectal Cancer Patients [32]. There are observational studies indicating that PTGS2 inhibitors could positively affect CRC patients’ survival after diagnosis [33–35]. NR3C2 is a mineralocorticoid receptor, also known as MR. Research has shown that MR can negatively regulate colorectal tumorigenesis. Besides, expression of MR is causally associated with the decrease of VEGFA expression, and VEGFA supports angiogenesis, which is critical for the growth and progression of solid tumors in a hypoxic environment [36]. CA 2 is highly expressed in different normal organs, but its expression is inhibited in cancer cells [37]. Besides, CA 2 overexpression can improve the CRC cells’ sensitivity to chemotherapy drugs, which may be a potential biomarker for early CRC diagnosis [38]. Research shows that the expression of MMP-1 in colorectal cancer tissues is higher than that in normal colon tissues, and MMP-1 may influence the occurrence and development of colorectal cancer through EMT and Akt signaling pathways [28]. In conclusion, the targets of the XCHT active ingredients interact with the disease targets of CRC, giving full play to the unique advantages of multi-compounds and multi-targets of traditional Chinese medicine to prevent and treat CRC.

After PPI network analysis, CCNB1 and SPP1 were defined as the critical genes. CCNB1, also known as cyclin B1, and high expression of CCNB1 may be associated with poor survival in patients with CRC [39]. The expression of SPP1 was up-regulated in CRC cells, and it may be a potential key target for the treatment of CRC [40].

Through GO enrichment analysis, it is found that the anti-CRC targets of XCHT mainly involve the regulation of neuroinflammatory response, collagen catabolic process, and embryo implantation to play the role of anti-CRC, which reflects the way of traditional Chinese medicine in the treatment of diseases. The results of the KEGG enrichment analysis suggest that besides the enrichment in cancer-related pathways, such as prostate cancer, bladder cancer. The targets of the main compounds of XCHT are also concentrated in tumors, signal pathways, and other pathways closely related to CRC, such as Prostate cancer, IL-17 signaling pathway, TNF signaling pathway, Toll-like receptor signaling pathway, NF-kappa B signaling pathway, etc. Relevant studies have shown that IL-17 inhibits CD8+ CTLs and Tregs’ infiltration by signaling in colorectal tumor cells, thereby promoting CRC development [41]. Tumor necrosis factor-α (TNF-α), a cytokine, and an essential inflammatory mediator, plays a vital role in malignant cellular proliferation, angiogenesis, tissue invasion, and metastasis in CRC [42]. Dysregulation of toll-like receptor (TLR) signaling pathway can result in disturbance of epithelial layer hemostasis, chronic inflammatory, excessive repair responses, and development of CRC [43]. NF-kappa B signaling pathway plays a key role in the cell proliferation, apoptosis, angiogenesis, and metastasis of CRC [44]. Besides, the targets of the main compounds of XCHT are also enriched in pathways related to inflammation, including Rheumatoid arthritis, Coronavirus disease COVID-19 etc., suggesting that XCHT may act on a variety of cytokines anti-inflammatory and have an effect on CRC.

According to the " Herb-Active ingredient-Target " network results, we selected 5 active ingredients and four key target genes to docking, and the docking results that these compounds could bind stably to the active pocket of the key protein, which indicates that XCHT may treat CRC by inhibiting PTGS2, NR3C2, CA2, MMP1.

From the perspective of network pharmacology, this study preliminarily elaborates on the potential active ingredients, possible targets, and critical biological pathways of XCHT in CRC treatment, providing a theoretical basis for further experimental verification. Given the limitations of network pharmacology, the pharmacological mechanism of XCHT in the treatment of CRC is only predicted by mining data, and the effects of the content of various compounds, the interaction between compounds and the metabolic process of herbs in vivo were ignored, which needs to be further verified through pharmacological and clinical studies.

## Conclusion

In summary, this study demonstrated the potential XCHT effector mechanism in treating CRC based on a network pharmacology method. We revealed that quercetin, stigmasterol, kaempferol, baicalein, and acacetin played a critical role in colorectal cancer by affecting PTGS2, NR3C2, CA2, and MMP1. Besides, our molecular docking studies also showed that the main active XCHT components (quercetin, stigmasterol, kaempferol, baicalein, and acacetin) could dock well with PTGS2, NR3C2, CA2, and MMP1, providing an important basis for further investigation. However, this study also has certain limitations as pharmacological and clinical research still need to further validate our findings.

## Supporting information

S1 Fig Results of molecular docking. (PDF)

S1 Table Basic information of active compounds in XCHT. (XLSX)

S2 Table Potential target gene of XCHT in the treatment of CRC. (XLSX)

S3 Table Top 20 potential gene targets and ingredients of XCHT. (XLSX)

S4 Table Enrichment analysis of GO function. (XLSX)

S5 Table Top 11 of enrichment analysis of KEGG function. (XLSX)
