# Supplementary material for: Network pharmacology and molecular docking study on the mechanism of colorectal cancer treatment using Xiao-Chai-Hu-Tang
Source: PLoS One. 2021 Jun 14;16(6):e0252508. doi: 10.1371/journal.pone.0252508 (PMC8202922; doi:10.1371/journal.pone.0252508)
Supplement: S1 Fig — (PDF) [file pone.0252508.s001.pdf]

S1 Fig.　Molecular docking Results

For Educational Use Only

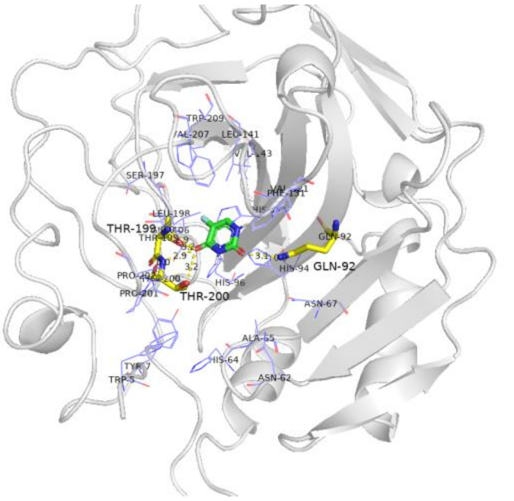

1. CA2 - 5-FU

For Educational Use Only

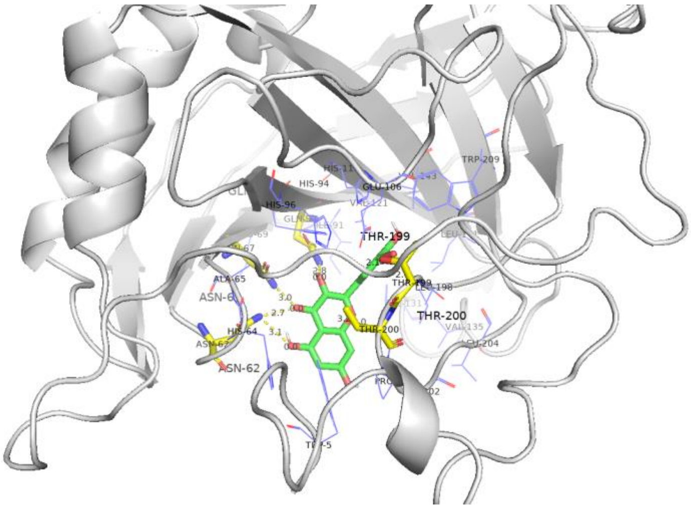

2. CA2 - MOL000098

For Educational Use Only

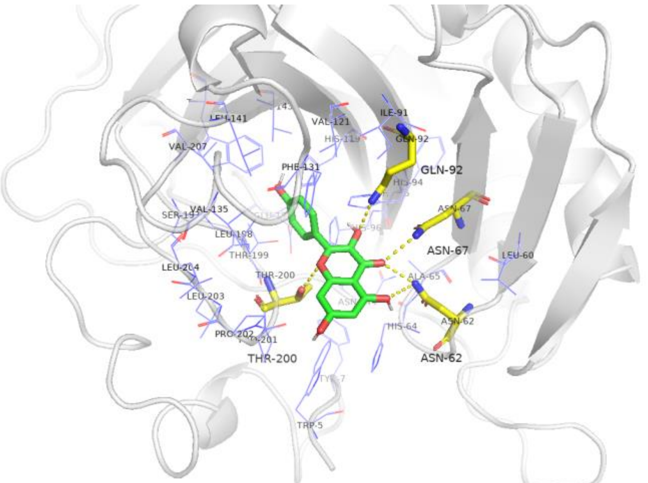

3.　CA2 - MOL000422

For Educational Use Only

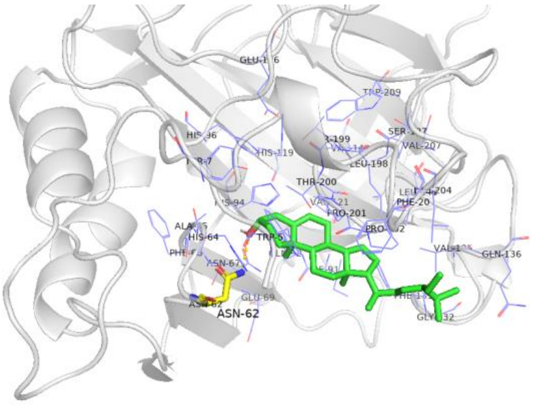

4. CA2 - MOL000449

For Educational Use Only

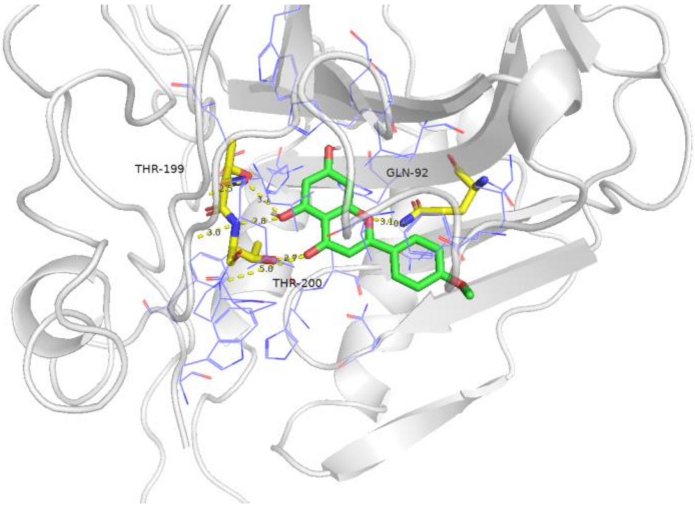

5. CA2 - MOL001689

For Educational Use Only

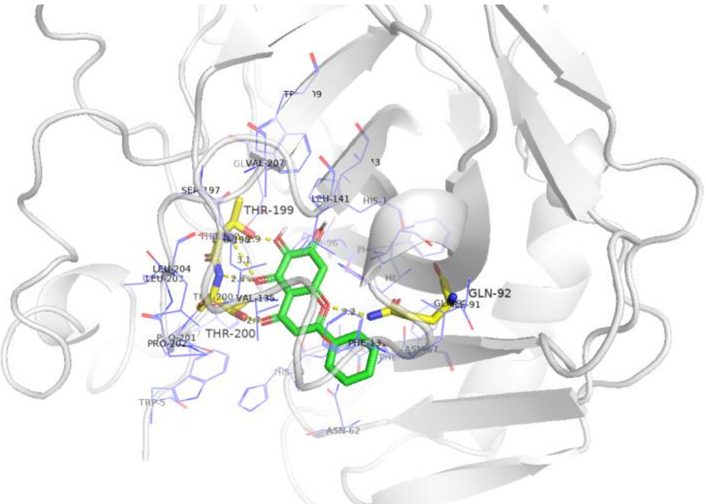

6. CA2 - MOL002714

For Educational Use Only

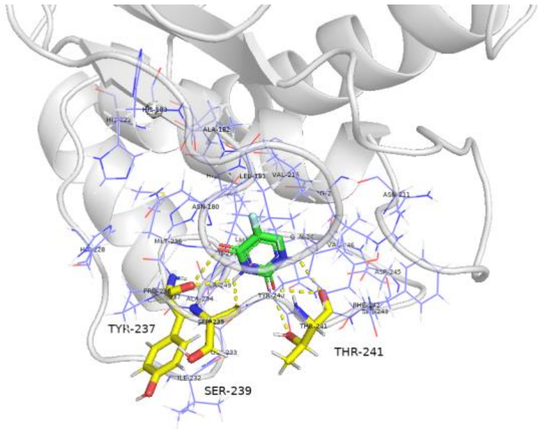

7. MMP1 - 5-FU

For Educational Use Only

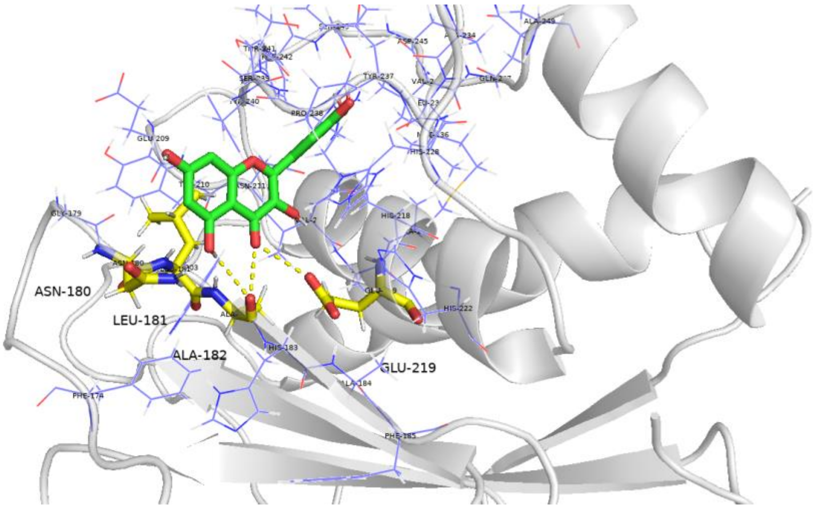

8. MMP1 - MOL000098

For Educational Use Only

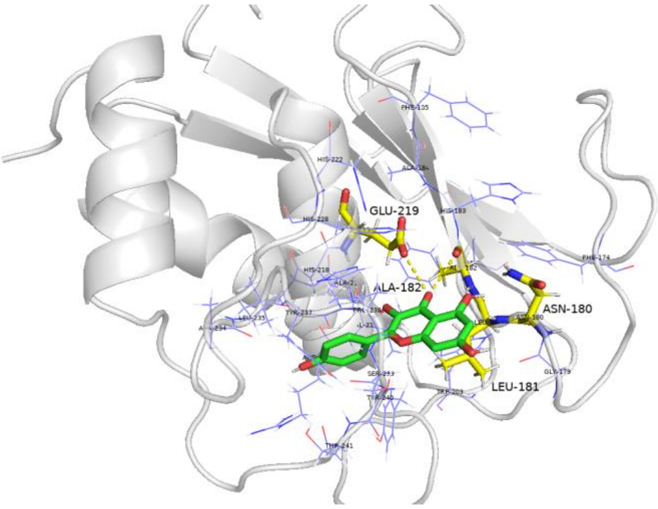

9. MMP1 - MOL000422

For Educational Use Only

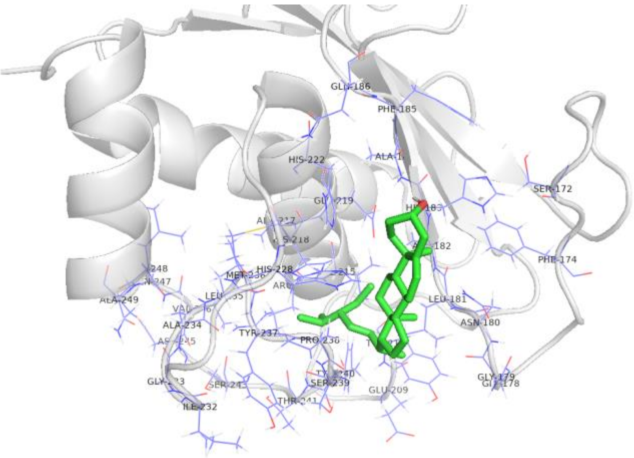

10. MMP1 - MOL000449

For Educational Use Only

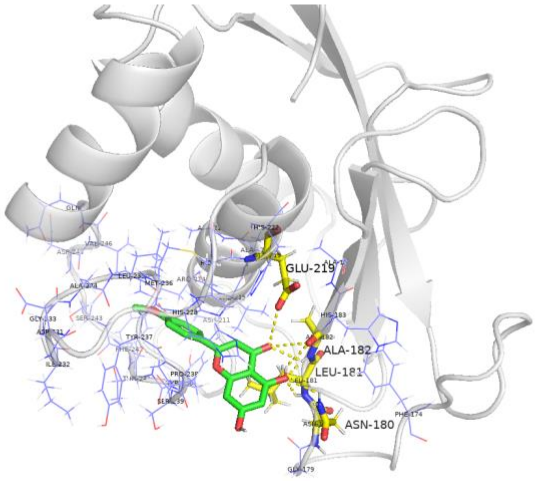

11. MMP1 - MOL001689

For Educational Use Only

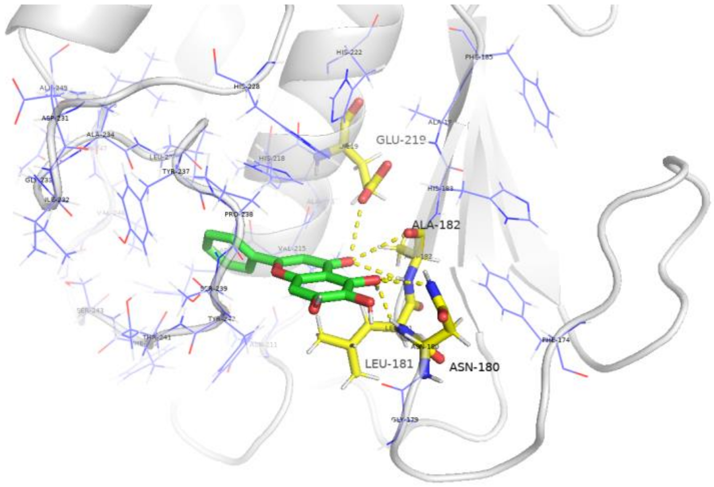

12. MMP1 - MOL002714

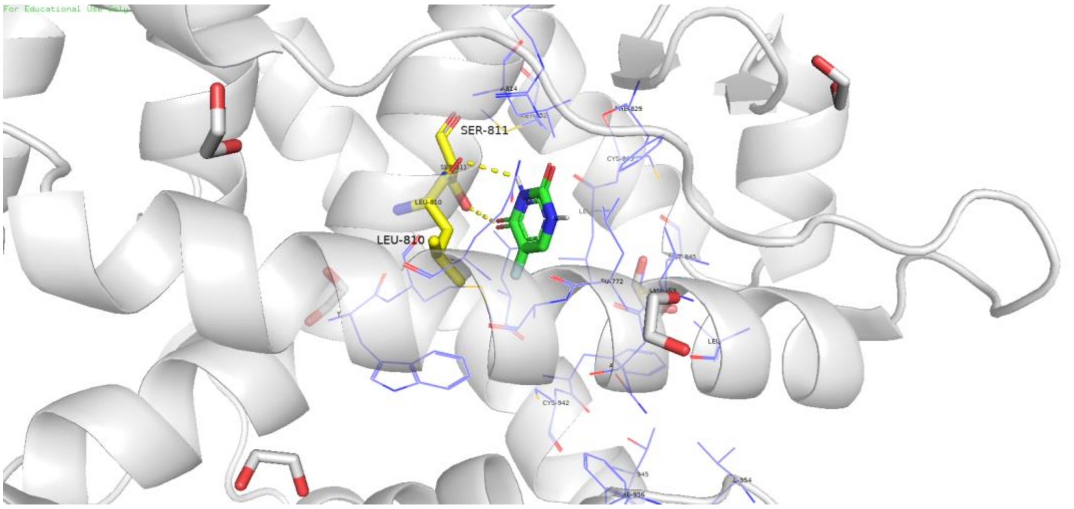

13. NR3C2 - 5-FU

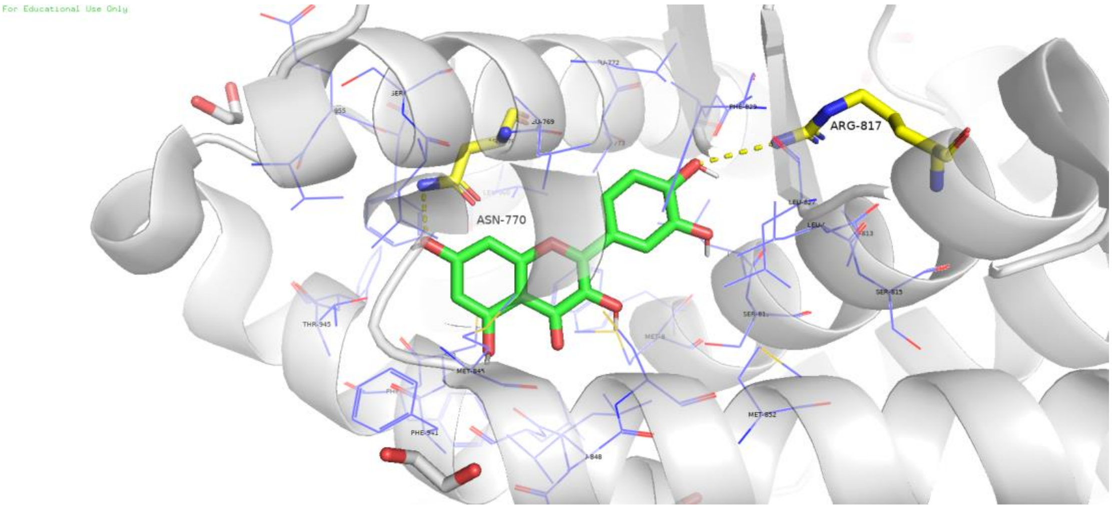

14. NR3C2 - MOL000098

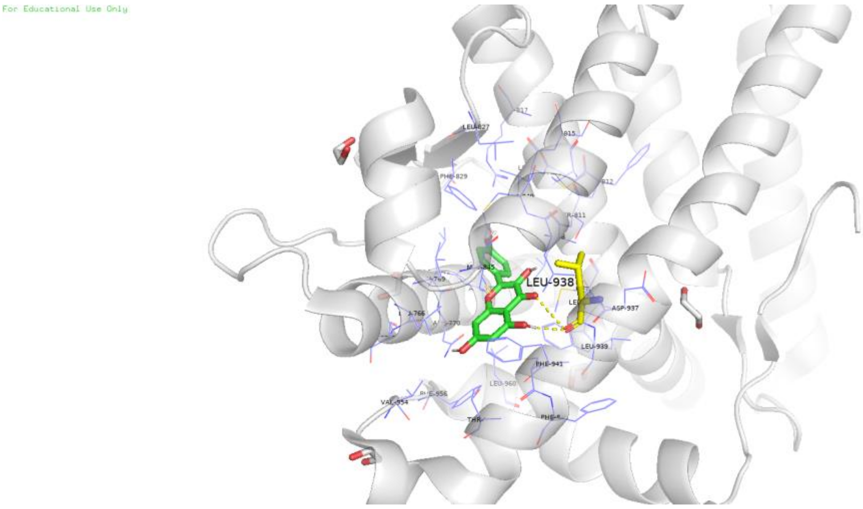

15. NR3C2 - MOL000422

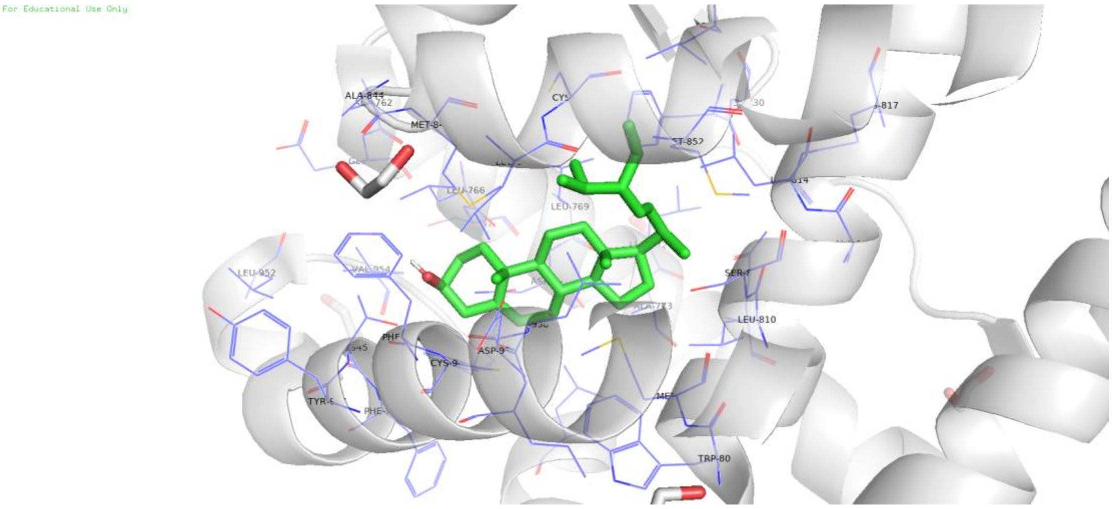

16. NR3C2 - MOL000449

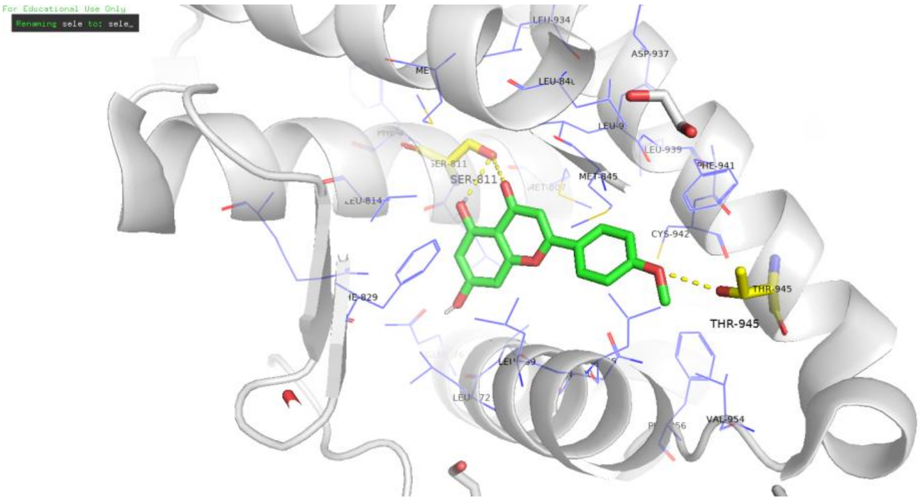

17. NR3C2 - MOL001689

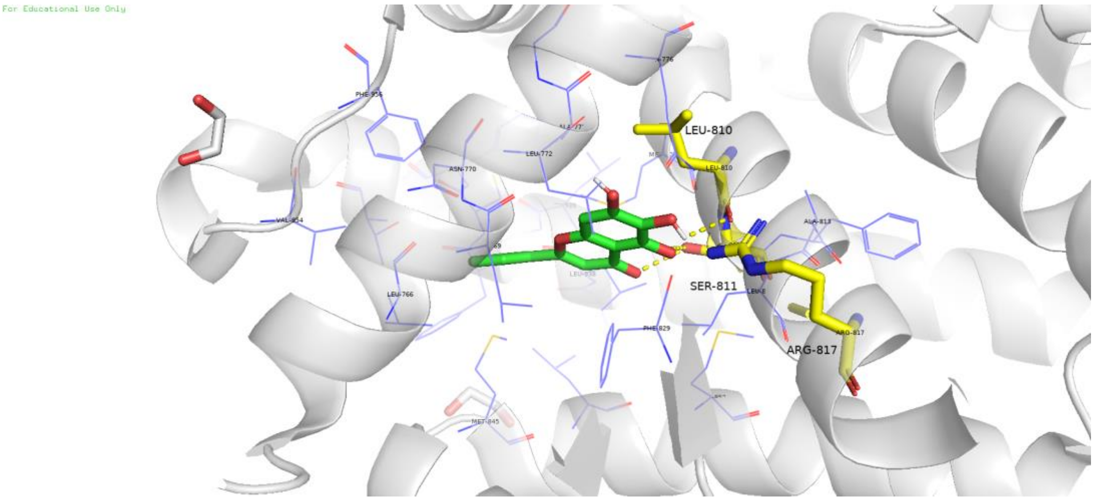

18. NR3C2 - MOL002714

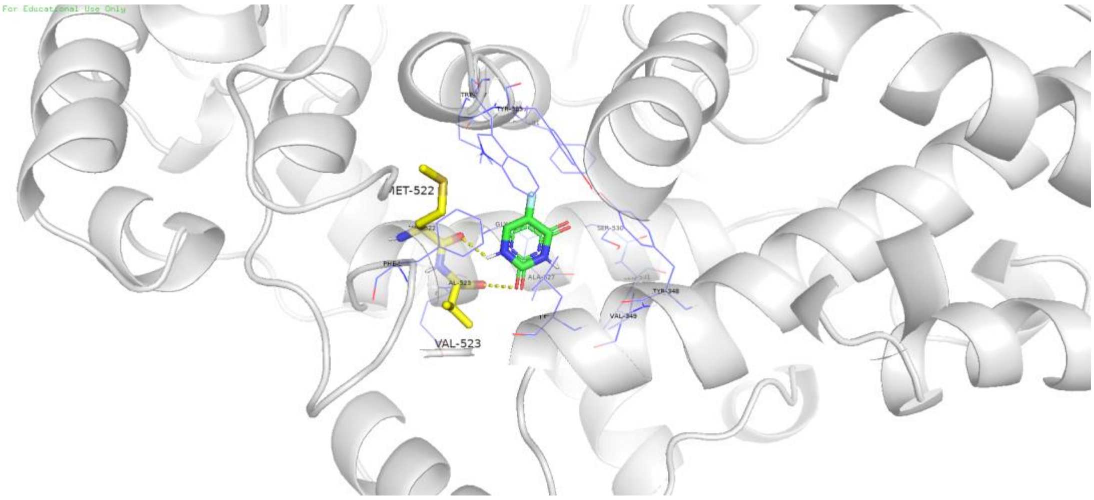

19. PTGS2 - 5-FU

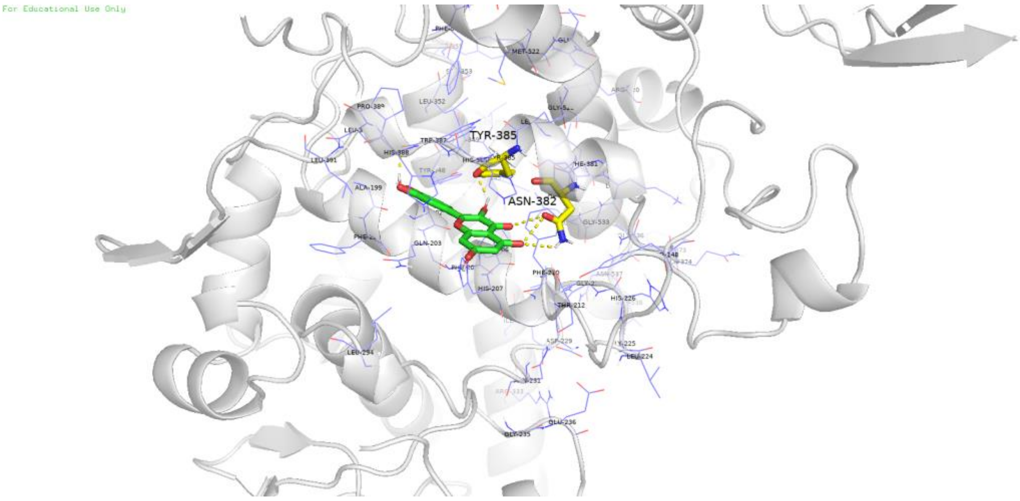

20. PTGS2 - MOL000098

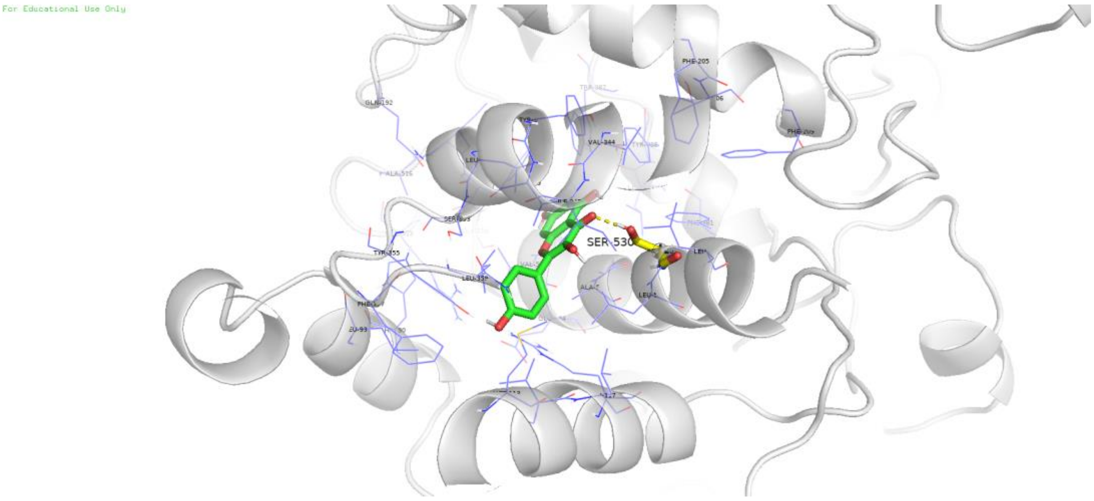

21. PTGS2 - MOL000422

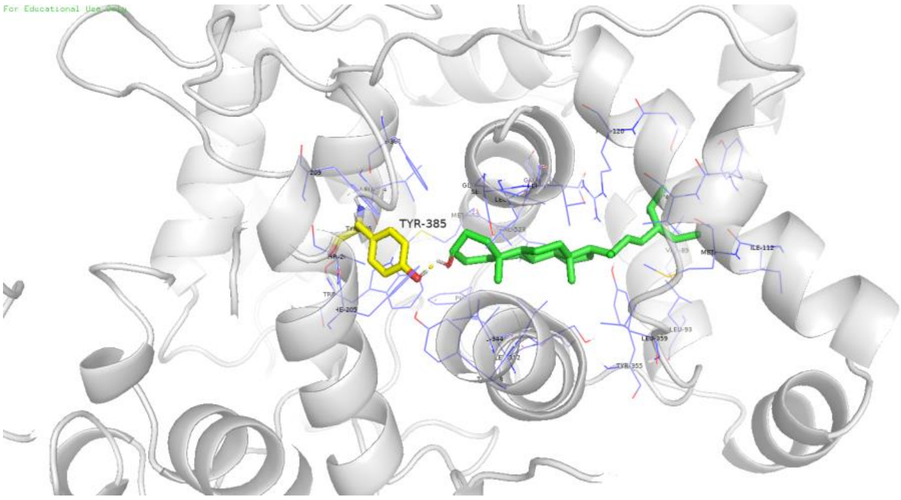

22. PTGS2 - MOL000449

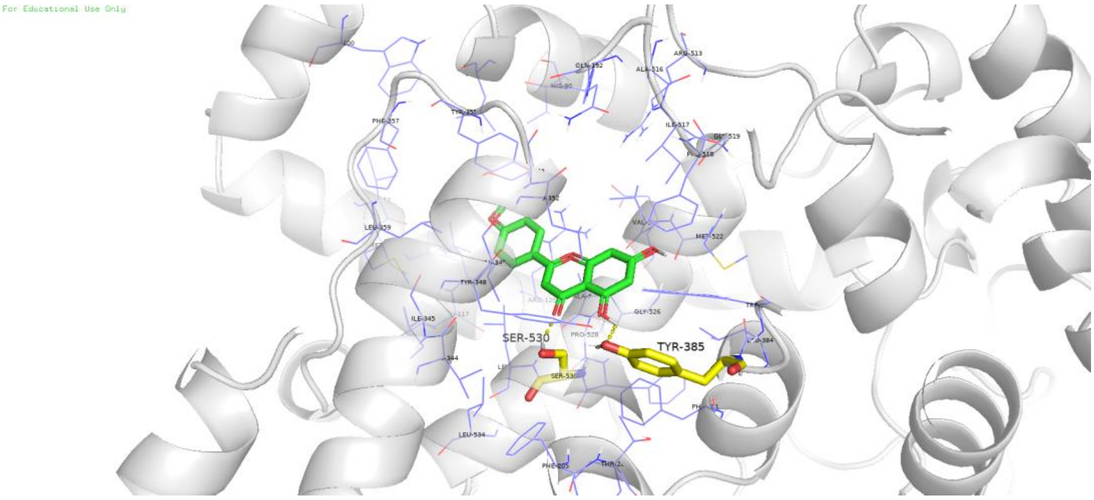

23. PTGS2 - MOL001689

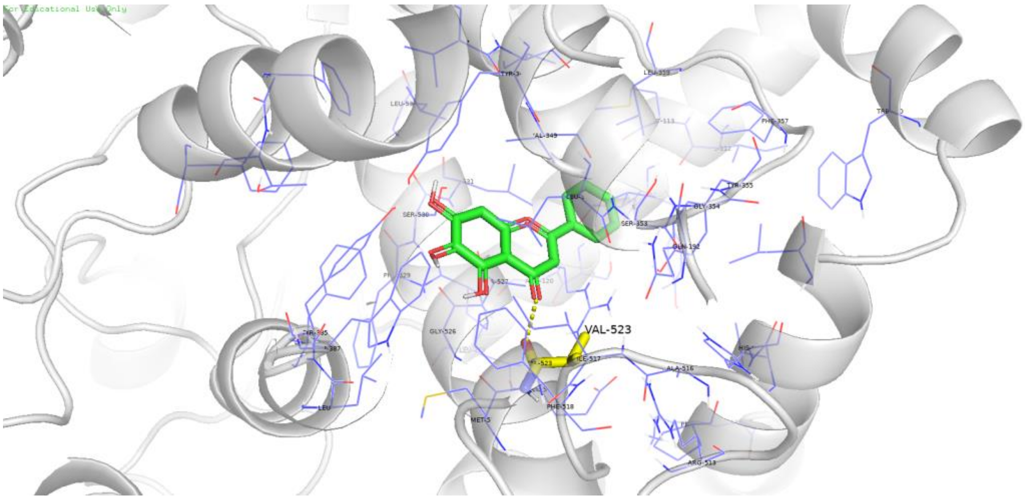

24. PTGS2 - MOL002714
